# A Report on Statistics of an Online Self-screening Platform for COVID-19 and Its Effectiveness in Iran

**DOI:** 10.34172/ijhpm.2020.252

**Published:** 2021-01-16

**Authors:** Sina Azadnajafabad, Sahar Saeedi Moghaddam, Negar Rezaei, Erfan Ghasemi, Shohreh Naderimagham, Mehrdad Azmin, Esmaeil Mohammadi, Kosar Jamshidi, Nima Fattahi, Hossein Zokaei, Ashkan Mehregan, Bahman Damerchilu, Pouya Fathi, Hossein Erfani, Abbas Norouzinejad, Mohammad Mehdi Gouya, Hamidreza Jamshidi, Reza Malekzadeh, Bagher Larijani, Farshad Farzadfar

**Affiliations:** ^1^Non-Communicable Diseases Research Center, Endocrinology and Metabolism Population Sciences Institute, Tehran University of Medical Sciences, Tehran, Iran.; ^2^Center for Communicable Diseases Control, Ministry of Health & Medical Education, Tehran, Iran.; ^3^School of Medicine, Department of Pharmacology, Shahid Beheshti University of Medical Sciences, Tehran, Iran.; ^4^Digestive Diseases Research Center, Digestive Diseases Research Institute, Tehran University of Medical Sciences, Tehran, Iran.; ^5^Endocrinology and Metabolism Research Center, Endocrinology and Metabolism Clinical Sciences Institute, Tehran University of Medical Sciences, Tehran, Iran.

**Keywords:** COVID-19, Self-screening, Epidemics, Iran, Public Health

## Abstract

**Background:** The most recent emerging infectious disease, coronavirus disease 2019 (COVID-19), is pandemic now. Iran is a country with community transmission of the disease. Telehealth tools have been proved to be useful in controlling public health disasters. We developed an online self-screening platform to offer a population-wide strategy to control the massive influx to medical centers.

**Methods:** We developed a platform operating based on given history by participants, including sex, age, weight, height, location, primary symptoms and signs, and high risk past medical histories. Based on a decision-making algorithm, participants were categorized into four levels of suspected cases, requiring diagnostic tests, supportive care, not suspected cases. We made comparisons with Iran STEPs (STEPwise approach to Surveillance) 2016 study and data from the Statistical Centre of Iran to assess population representativeness of data. Also, we made a comparison with officially confirmed cases to investigate the effectiveness of the platform. A multilevel mixed-effects Poisson regression was used to check the association of visiting platform and deaths caused by COVID-19.

**Results:** About 310 000 individuals participated in the online self-screening platform in 33 days. The majority of participants were in younger age groups, and males involved more. A significant number of participants were screened not to be suspected or needing supportive care, and only 10.4% of males and 12.0% of females had suspected results of COVID-19. The penetration of the platform was assessed to be acceptable. A correlation coefficient of 0.51 was calculated between suspected results and confirmed cases of the disease, expressing the platform’s effectiveness.

**Conclusion:** Implementation of a proper online self-screening tool can mitigate population panic during wide-spread epidemics and relieve massive influx to medical centers. Also, an evidence-based education platform can help fighting infodemic. Noticeable utilization and verified effectiveness of such platform validate the potency of telehealth tools in controlling epidemics and pandemics.

## Background

**Key Messages**

** Implications for policy makers**

- Population panic during an epidemic is a significant public health problem leading to a massive influx to medical centers, and this panic needs proper policies to be controlled.
- Population-wide screening strategies during an epidemic is an essential aspect of disaster management.
- Ongoing infodemic besides the real epidemic adds to the burden of the disease in communities; therefore, health systems should implement proper instructions to fight the infodemic effectively.
- Preparing populations and health systems for the next waves of the current coronavirus disease 2019 (COVID-19) epidemic and similar other epidemics and pandemics is a necessary step that governments can test policies right now within the epidemic.

** Implications for the public**

The results of this study can be used to investigate the nature and transmission of the epidemic in Iran. Successful implantation and proven potency of such platform suggest more application of telehealth in public health disasters. Details of the platform in this study can be useful for further deployment of similar platforms. Any population-wide survey about coronavirus disease 2019 (COVID-19) and its characteristics, especially self-screening and symptom studies, can be compared to this study’s results. Evidence-based medical education can help people learn the proper instructions and more effectively cope with the frightening condition of the epidemic. Informed people even can help governments and health systems to more efficiently conduct the protocols of controlling the disease.

Emerging infectious diseases are complex public health concerns affecting populations and governments.^1^ The most recent example, coronavirus disease 2019 (COVID-19) caused by the novel coronavirus named SARS-CoV-2 (severe acute respiratory syndrome coronavirus 2) today, first was seen in Wuhan, China, but has vastly spread almost in all countries globally.^2^ This enormous spread made the World Health Organization (WHO) declare the COVID-19 pandemic on March 11, 2020; in less than 3 months of the epidemic’s beginning in China.^3^ Iran, placed in the Middle East region, reported the first confirmed case of COVID-19 on February 19, 2020, from Qom city.^4^ As of May 12, 2020, Iranian officials reported a total of 110 767 confirmed cases of COVID-19, of which 6733 expired, and 88 357 recovered from the condition.^5^

 Hence this outbreak crisis does impose a heavy burden on medical systems by exceeding the capacity of hospitals all around the world, causing a shortage of both medical resources and healthcare professionals; standard communicative practices cannot handle the massive influx of patients to medical centers.^6^ One important factor causing such rushes in communities is people’s knowledge and attitudes toward COVID-19; therefore, health education programs improving the knowledge can help handle the crisis.^7^ Like other countries, Iran started different action plans to control the epidemic. Still, various obstacles like inadequate health infrastructure and medical resources lead to the widespread distribution of infection in the country.^8^ Thus, more efficient programs and policies are needed to tackle this situation in Iran and other countries.

 Previous studies explained the capability of telemedicine in various disasters and public health emergencies like epidemics.^9,10^ One of the well-described strategies named “forward triage,” that is categorizing patients based on their symptoms before they arrive in the medical centers, can be utilized through telemedicine to tackle disasters like COVID-19.^9^ Therefore COVID-19 epidemic created an excellent opportunity for health systems to develop and extend their telemedicine features to reduce the number of frightened well or low-risk people with minimal symptoms visiting medical centers.^11^ Similar studies have been done on populations by asking about demographic characteristics, traveling history, relevant symptoms, previous medical history and conditions, and preliminary physical examinations like body temperature to expand the application of telemedicine in controlling the coronavirus epidemic.^12^ Since physical symptoms and sign screenings are potentially limited in effectiveness in epidemics, due to many recently exposed and asymptomatic cases,^13,14^ we can trust online screenings and telehealth services as a complementary tool to screen suspected cases. Since there is no definite treatment and vaccine for this disease yet, we should carefully handle suspected and confirmed cases, and a registry system for all patients’ documents and samples is necessary to facing more efficiently possible future similar epidemics.^15,16^

 The aim of this study was the introduction and assessment of an online self-screening platform for symptoms of COVID-19 and similar conditions like influenza and the cold, which could provide necessary instructions and educations for people at home and before visiting a physician in Iran. Such an efficient system could prevent unnecessary visits in medical facilities, places which are overcrowded these days not only by COVID-19 suspected cases but also with panicked healthy people seeking an explanation for their symptoms and possible examinations and tests. Giving people the proper knowledge and information about the disease would reduce this anxiety and misperceptions about the recent pandemic.

## Methods

###  Team Formation and Data Collection Platform Development

 In less than 1 week after the report of the first positive case of COVID-19 in Iran, we gathered a multidisciplinary team composed of epidemiologists, physicians, biostatisticians, data science experts, and information technology technicians to initiate the platform. This team was originated in Non-Communicable Diseases Research Center, a research institute of Endocrinology and Metabolism Research Institute affiliated to Tehran University of Medical Sciences, supported by Deputy of research and technology of Ministry of Health and Medical Education (MoHME) of Iran. We developed an online framework (https://corona.research.ac.ir/) in the Persian language, with 3 primary functions: First, translated evidence-based educational content acquired from globally authorized institutions like WHO, Centers for Disease Control and Prevention (CDC), and National COVID-19 Epidemiology Committee of Iran^17-19^ were prepared and published on the website to make sure people receive the proper and essential contents on the epidemic; Second, a through registry system for suspected and positively confirmed COVID-19 patients, that enrollments are taking place in medical centers; Third, a concise self-screening system that decides to give instructions to help-seekers based on their symptoms and background diseases by an algorithm, which is the main goal of this study.

###  Datasets

 Four main datasets were utilized in this study. The main dataset was the participants’ submitted self-screening data in the mentioned platform. To compare the representativeness of the participated population in this platform, data of the WHO STEPwise approach to Surveillance (STEPS) of non-communicable diseases in Iran 2016 and national data from the Statistical Center of Iran were employed.^20,21^ To compare this tool’s effectiveness, data of the COVID-19 registry dataset by MoHME of Iran was considered (unpublished data). The registry dataset consisted of patients with confirmed positive laboratory results for COVID-19, having tests during admission in hospitals or during outpatient visits. This data was collected by the Deputy of health of MoHME and its deputations in all hospitals and medical centers.

###  Data Processing

 The primary dataset consisted of 440 638 participants. Duplicated observations were detected based on the browser name (Chrome, Firefox, Opera, Web Kit, Internet Explorer, Microsoft Edge, and Safari), browser version, device type (computer, mobile, and tablet), name of the operating system (Android, Linux, IOS, Windows, Mac OS X, Chrome OS, Ubuntu, and Tizen), internet protocol (IP) address, and the date of visiting the platform. Also, observations that had filled the same result during different visiting times were removed. We dropped participants with body mass index (BMI) <10 kg/m^2^ or BMI >80 kg/m^2^ as the implausible BMI range among 14+ years old.^22^ The ultimate dataset contained 309 648 participants.

###  Self-screening Algorithm

 The questions asked in this online survey consists of gender, age, weight (in kilograms), height (in meters), location (province), series of primary symptoms (dry cough or chill or sore throat), dyspnea (shortness of breath), body temperature (in Celsius), and high risk past medical histories categorized in 2 groups of the immunodeficient group including receiving corticosteroids, history of transplantation, chemotherapy, cancer, HIV/AIDS (human immunodeficiency virus/acquired immune deficiency syndrome), and underlying diseases including cardiovascular diseases, hypertension, chronic respiratory diseases, and diabetes mellitus. BMI ≥ 40 kg/m^2^ and age ≥50 years-old are also assumed as high-risk conditions. Body temperatures equal to or greater than 37.8°C are defined as fever.

 We developed an algorithm for self-screening, which codes exhibited in Table 1, inspired by the confirmed algorithm of patients management published by MoHME of Iran.^23^ Based on the submitted information, participants receive 4 levels of guidance for their history: The first level are suspected cases of COVID-19 and are referred to designated centers for the condition for possible admission and diagnostic tests, and a map of these centers is provided online to help patients. In the second level, patients are suggested to visit the nearest medical center to do more diagnostic laboratory and radiologic tests and even admission, based on the severity of their symptoms and background conditions and a map of nearest medical centers are provided for them in the results part. In the third level patients are suggested to be more careful, take supportive care at home and do reassessment daily or in the case of changing symptoms. Participants that are not categorized in 3 previous levels are placed in the fourth level and are suggested to consider preventive measures. For all 4 types of patients, educational content relevant to their condition is also provided on the result page, like prevention and isolation measures.

**Table 1:** Self-screening Algorithm Installed on the Website

**Number**	**Code **	**Function (Suggestion to the Participant)**
1	(primary symptoms positive and dyspnea positive) *or* (primary symptoms negative and dyspnea positive and bodytemperature ≥37.8)	You are suspected Refer to designated centers for COVID-19
2	(primary symptoms positive and dyspnea negative and body temperature ≥37.8 and (immunodeficient group positive or underlying disease positive or age ≥50 or BMI ≥40)) *or* (primary symptoms negative and body temperature ≥ 37.8 and (immunodeficient group positive or underlying disease positive or age ≥ 50 or BMI ≥40)) *or* (primary symptoms negative and body temperature ≥37.8 and dyspnea positive and (immunodeficient group positive or underlying disease positive or age ≥50 or BMI ≥40))	You should visit the nearest medical center to do more diagnostic tests
3	(primary symptoms positive and dyspnea negative and body temperature <37.8) *or * (primary symptoms positive and dyspnea negative and body temperature ≥37.8 and underlying disease negative and immunodeficient group negative) *or* (primary symptoms negative and dyspnea negative and body temperature ≥37.8 and (underlying disease negative and immunodeficient group negative and age <50 and BMI <40))	You should be more careful Consider supportive care and do a reassessment
4	None of the conditions above fulfilled	You are not suspected Consider preventive measures

Abbreviations: COVID-19, coronavirus disease 2019; BMI, body mass index.

###  Online Platform Infrastructure

 The first date of website deployment was on February 25, 2020, and the first public access started on March 3, 2020. Distribution was done through social media and Iranian national news. We developed the platform on a Java-based Spring Framework. The implementation process was done by RABIT (Research and Business Integrated Tools) engine system,^24^ which composed of 3 subsystems of DIGIT (Design, Implementation, and Gathering data through Integrated Tool) that helps to design and implement electronic surveys, registries, and evaluation systems,^25^ VIZIT (Visualization Integrated Tool) that is a visualization engine to generate dynamic reports from data,^26^ and Sumit which is an online data pipeline that provides analytical application programming interfaces.^27^

###  Statistical Analysis

 Pearson’s correlation coefficient was used to measures the linear correlation between suspected participants and different conditions (primary symptoms, the experience of dyspnea, fever, and underlying diseases) based on the positive laboratory results in the COVID-19 registry. Also, we reported Pearson’s correlation coefficient to check the linear correlation between the prevalence of underlying diseases among the general population of 15 years-old and more with positive laboratory results in the COVID-19 registry. Multilevel mixed-effects Poisson regression was used to check the association of visiting platform and death from the COVID-19 registry after adjusting by age, sex, and the covariates since the analyzed data had a hierarchical structure. In this regard, successful years of schooling and wealth index – extracted from household income and expenditure survey– and urbanization – extracted from population and housing census – by Statistical Center of Iran were applied as covariates. A model with the lowest Akaike information criterion was considered to have the best fit.^28^ All statistical analyses were calculated with the STATA software version 14 (StataCorp, Texas, USA). Figures were depicted by R software version 3.0.2 (Vienna, Austria).

## Results

###  Descriptive Report of Participants

 Records of online self-screenings in the first 33 days of platform utilization included about 310 000 unique and reliable submissions. Primary symptoms were positive in 34.9% (95% CI: 34.7 to 35.2) of males and 41.0% (95% CI: 40.7 to 41.2) of females. Dyspnea as a major symptom was present in 20.7% (95% CI: 20.5 to 20.9) of males and 22.2% (95% CI: 21.9 to 22.4) of females. 10.5% (95% CI: 10.3 to 10.6) of males and 8.1% (95% CI: 7.9 to 8.2) of females had fever on body temperature reports. 17.4% (95% CI: 17.3 to 17.6) of male participants had a history of underlying diseases, and this number was 16.8% (95% CI: 16.6 to 17.0) in females. 12.6% (95% CI: 12.4 to 12.7) of male and 12.0% (95% CI: 11.8 to 12.2) of female participants were categorized as high-risk age groups. A complete report of statistics of participants and recorded conditions are provided in Table 2. All reported differences in both genders were statistically significant. (*P* value <.001, statistical analysis by *t* test).

**Table 2:** Distribution of Positive Reported Conditions on Online Self-screening Submissions Stratified by Sex

**Condition**	**Gender**				***P*** ** Value**
	**Male**		**Female**		
	**N**	**% (95% CI)**	**N**	**% (95% CI)**	**<.001**
Primary symptoms (chill, sore throat, or dry cough)	60 988	34.9 (34.7 to 35.2)	55 330	41.0 (40.7 to 41.2)	<.001
Experience of dyspnea	36 164	20.7 (20.5 to 20.9)	29 920	22.2 (21.9 to 22.4)	<.001
Fever (body temperature ≥ 37.8°C)	18 262	10.5 (10.3 to 10.6)	10 922	8.1 (7.9 to 8.2)	<.001
Underlying diseases (CVDs, HTN, CRD, or DM)	30 457	17.4 (17.3 to 17.6)	22 638	16.8 (16.6 to 17.0)	<.001
Immunodeficiency (chemotherapy, HIV/AIDS, transplantation)	5514	3.2 (3.1 to 3.2)	4946	3.7 (3.6 to 3.8)	<.001
Morbid obesity (BMI ≥40 kg/m^2^)	2989	1.7 (1.6 to 1.8)	2678	2.0 (1.9 to 2.1)	<.001
High-risk age groups (age ≥50 years old)	22 000	12.6 (12.4 to 12.8)	16 203	12.0 (11.8 to 12.2)	<.001

Abbreviations: CVDs, cardiovascular diseases; HTN, hypertension; CRD, chronic respiratory disease; DM, diabetes mellitus; HIV, human immunodeficiency virus; AIDS, acquired immune deficiency syndrome; BMI, body mass index. *Note: t* test was used to investigate reported *P* values in this table.

**Figure 1:**
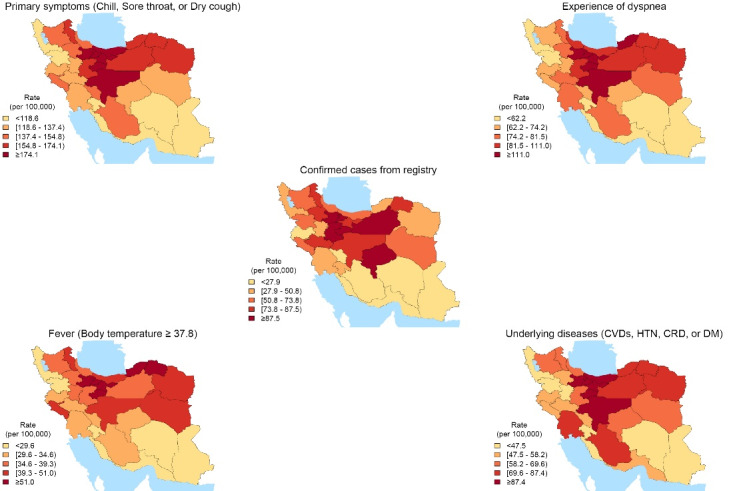
The Pattern of Primary Symptoms, Dyspnea, Fever, Underlying Diseases From the Submitted Information in the Self-screening Platform, and Confirmed Cases From the Registry of COVID-19 at the Subnational Level. Distribution of signs and symptoms and confirmed cases represent distinct variations. Abbreviations: CVDs, cardiovascular diseases; HTN, hypertension; CRD, chronic respiratory disease; DM, diabetes mellitus; COVID-19, coronavirus disease 2019.

 Comparing the provincial distribution of rates of symptoms and underlying diseases with confirmed cases of COVID-19 from the registry revealed different patterns in Iran. Among the mentioned conditions fever had a more diverse pattern compared to others. Central provinces of Iran had both the most rates of reported symptoms and positive cases. Patients in areas with higher rates of underlying diseases also experienced higher rates of primary symptoms and dyspnea. (Figure 1).

 The mean age of all included participants was 34.87 (standard deviation: 13.06), with a range of 14-114 and a median of 33.00. The mean age of male participants was 35.3 (95% CI: 35.2 to 35.4) versus 34.3 (95% CI: 34.2 to 34.4) mean age of females. Mean BMI was 26.55 (95% CI: 26.53 to 26.58) and 25.77 (95% CI: 25.74 to 25.80) in males and females, respectively. Overall mean submitted body temperature was higher in suspected and diagnostic tests needing groups. Mean age, BMI, and body temperature were statistically higher in the group needed further diagnostic tests (*P* <.001) (Table 3).

**Table 3:** Mean Age, BMI, and Body Temperature (Celsius) in 4 Levels of Final Results of Self-screening

**Variable**	**Suspected**	**Diagnostic Tests**	**Supportive Care**	**Not Suspected**	***P*** ** Value**
Age	35.55 (35.39 to 35.72)	45.66 (45.12 to 46.19)	33.52 (33.45 to 33.60)	35.13 (35.07 to 35.19)	<.001
BMI	26.51 (26.44 to 26.58)	28.61 (28.39 to 28.83)	25.98 (25.94 to 26.01)	26.21 (26.19 to 26.24)	<.001
Body temperature	37.06 (37.04 to 37.07)	38.30 (38.28 to 38.31)	36.73 (36.72 to 36.74)	36.31 (36.30 to 36.31)	<.001

Abbreviation: BMI, body mass index. *Note*. Comparisons were made by *t* test to produce *P* values.

 Final 4 level of results categorized by gender and age groups shows that most of the participants were diagnosed not a COVID-19 suspect in self-screening. Only 10.4% of males and 12.0% of females were diagnosed suspected of COVID-19. Age groups 20-29 and 30-39 had the highest proportion of participants, but age groups of 60 years-old and more had the highest ratio of suspected (18.0%) and suggested doing necessary diagnostic tests (6.6%) among age groups. (Table 4).

**Table 4:** Details of Final Self-screening Results Based on Submitted Information on the Platform

		**Suspected**	**Diagnostic Tests**	**Supportive Care**	**Not Suspected**	***P*** ** Value**
Gender	Male	18 239 (10.4%)	2814 (1.6%)	47 857 (27.4%)	105 706 (60.5%)	<.001
	Female	16 154 (12.0%)	1836 (1.4%)	41 674 (30.9%)	75 368 (55.8%)	
Age groups	14-19	3275 (11.4%)	341 (1.2%)	7688 (26.7%)	17 465 (60.7%)	<.001
	20-29	10 109 (12.4%)	690 (0.8%)	26 077 (32.1%)	44 381 (54.6%)	
	30-39	11 633 (10.3%)	860 (0.8%)	35 206 (31.2%)	65 203 (57.8%)	
	40-49	4460 (9.2%)	683 (1.4%)	12 880 (26.5%)	30 494 (62.9%)	
	50-59	2025 (9.1%)	1017 (4.6%)	4639 (20.9%)	14 501 (65.4%)	
	60 years and more	2891 (18.0%)	1059 (6.6%)	3041 (19.0%)	9030 (56.4%)	

*Note*. Results of 4 categories of results, including suspected cases, requiring diagnostic tests, supportive care, and not suspected cases produced by the algorithm deployed in the online platform, are presented stratified by sex and age groups of study. (Comparisons between 4 levels of results were made by chi-square test).

**Figure 2:**
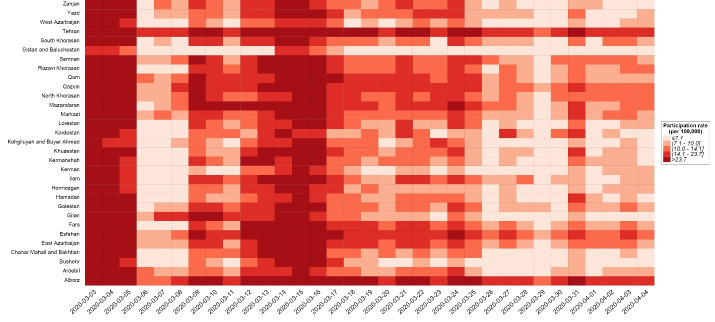
Age-Standardized Rate of Online Self-screenings During 1 Month of Platform Operation in 31 Provinces of Iran. This heatmap represents the density of self-screenings that happened each day in each province. As the color of the map gets darker, more screenings took place on that day and location on the map.

 Distribution of age-standardized rate of recorded online self-screenings among 31 provinces of Iran shows diverse patterns in different locations (Figure 2). Highly affected provinces like Tehran, Alborz, and Isfahan had the highest rates of online self-screening. Submissions happened more in the first 3 weeks after the introduction of the platform. According to a multilevel mixed-effects Poisson regression model showing the impact of deaths caused by the COVID-19 adjusted by other covariates on the use of online screenings based on locations, for each confirmed death due to COVID-19 happened, 0.3 more online self-screening submitted, in 100 population (Table 5).

**Table 5:** Multilevel Mixed-Effects Poisson Regression Assessing the Association of Platform Submission and Death From COVID-19, Adjusted by Covariates

	**Coefficient**	**SE**	***P*** ** > |z|**	**95% CI**	
Death/100	0.30	0.15	.046	0.01	0.60
Gender/Male	0.19	0.05	.000	0.10	0.28
Age group					
20 to 24	0.43	0.11	.000	0.20	0.65
25 to 29	0.80	0.11	.000	0.58	1.02
30 to 34	1.08	0.11	.000	0.87	1.28
35 to 39	1.04	0.11	.000	0.83	1.24
40 to 44	0.54	0.13	.000	0.29	0.79
45 to 49	0.05	0.15	.722	-0.24	0.35
50 to 54	-0.20	0.17	.249	-0.53	0.14
55 to 59	-0.62	0.20	.002	-1.02	-0.23
60 to 64	-0.86	0.23	.000	-1.32	-0.40
65 to 69	-1.34	0.27	.000	-1.87	-0.81
70 to 74	-1.88	0.33	.000	-2.52	-1.24
75 to 79	-2.46	0.39	.000	-3.22	-1.70
80 to 84	-2.80	0.44	.000	-3.66	-1.94
≥ 85 years	-2.36	0.37	.000	-3.08	-1.63
Wealth index	-0.11	0.10	.269	-0.31	0.09
Years of schooling	0.07	0.02	.006	0.02	0.12
Urbanization	0.58	0.90	.519	-1.18	2.34

Abbreviations: COVID-19, coronavirus disease 2019; SE, standard error.

###  Demographic Data in Comparison With Iran STEPs Study and Data Form Statistical Center of Iran

**Figure 3:**
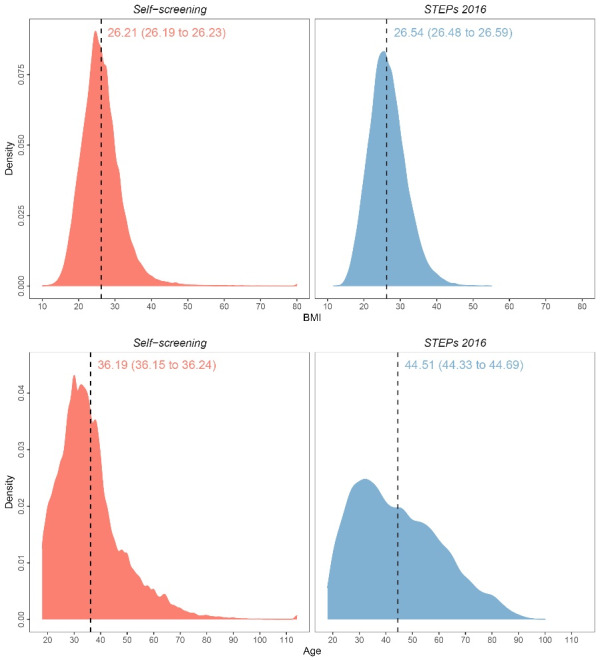
Age and BMI Density Plots With Mean and 95% CI Values in Self-screening and Iran STEPs 2016 Studies. Abbreviation: BMI, body mass index.

 In visualization and collation of age and BMI data in self-screening and Iran STEPs 2016 datasets (as a representative sample of Iranian adults), the mean BMI of all participants in this study was 26.21 (26.19 to 26.23), versus 26.54 (26.48 to 26.59) in STEPs 2016. Meanwhile, the mean age of participants ≥18 years-old (due to comparison with population included in STEPs study) in this study was 36.19 (36.15 to 36.24) versus 44.51 (44.33 to 44.69) in STEPs 2016 (Figure 3). Based on the estimated features of the Iranian population by the Statistical Center of Iran in 2019, age group 30-39 comprised the majority of the population (26.6%), as well as this study (36.6%). Also, in the same estimation, male gender was the dominant gender in the population (50.4%), as well as the present study (56.4%).

###  Platform Power of Differentiation

 Evaluation of correlations between confirmed COVID-19 cases by positive laboratory results and suspected level of the self-screening platform among participants ≥14 years old showed a fine correlation coefficient of 0.51. Also, the estimation of correlation between confirmed cases and positive primary symptoms, the experience of dyspnea, and the presence of fever revealed similar correlation coefficients of 0.51, 0.52, and 0.54, respectively.

## Discussion

 This study mainly demonstrated the more prominent distribution of different symptoms, signs, and various underlying conditions in central and northern provinces. Male users and younger age groups were more involved in the self-screening program. Reported data finely was representative of the general population of Iran that had access to online devices, based on gender and age distribution. This platform could effectively differentiate various conditions and histories, and final instructions were delicately appropriate according to confirmed cases (correlation coefficient of 0.51). An appropriate education platform can help to fight the associated infodemic during epidemics.

 A more detailed evaluation of results extracted from the self-screening platform introduced in this study provides us its beneficial features. Considering patterns of distribution of different signs and symptoms of the disease at the subnational level reveal a more prominent pattern and higher rates of self-report in central provinces, where the first cases of COVID-19 were reported and near provinces, mainly Tehran due to containing the capital city of the country, that lead to further transmission to other provinces like northern ones. The majority of participants were in younger age groups (20-29 and 30-39), probably mainly due to more access to social media and online devices and more willingness to use internet-based services, including health services. More than half of the participants were diagnosed not a suspect for COVID-19, and almost 9 out of 10 who submit their history were categorized as the 2 low-risk levels of the final screening results, which shows the importance and necessity of palliating public panic during such an epidemic.

 Comparing data originated from the self-screening platform with a similar national-wide study (Iran STEPs 2016, and Iran population estimation) displayed similar characteristics that bring up the point that platform has been well-distributed in different populations of the country and its penetration is noticeable. Equity and equality in access to health services are the benefits of telehealth that makes resources available for a more significant number of people.^29^ Online self-screenings happened mainly in the first 14 days after the implementation of the platform. After that, submissions diminished, mostly because the national online self-screening program started to run by MoHME about ten days after our website’s introduction.^30^ However, provinces like Tehran, Alborz, and Esfahan with higher COVID-19 contamination had a steadier self-screening pattern.

 Noteworthy correlations between the suspected level of results and confirmed cases of COVID-19 by laboratory results were the evidence for the effectiveness of this platform. The power of the platform to differentiate multiple combinations of histories, including numerous symptoms, signs, and underlying diseases, and to instruct final advice, is another benefit of such telehealth tools that can continuously operate and handle a great number of participants in a short time.

 During any emerging epidemic, the disease outbreak accompanies another outbreak of rumors and misinformation, called infodemic.^31^ This wrong information can have a substantial impact on people’s attitudes and behaviors, leading to the neutralization of governments’ action plans and policies to stop the outbreak,^32^ especially in the presence of social media, the infodemic disaster is expanded much faster. Probably an effective strategy is seeking help from health system authorities to spread honest and evidence-based information.^33^ As an education part of our platform, we published updated data and instructions based on global officials like WHO and tried to spread appropriate information about the epidemic. We provided content for distinct aim groups and tried to enrich people with concise and essential information. It is evident that the right knowledge is associated with positive attitudes, so health education programs improving knowledge will lead to more safe actions.^7^

 COVID-19 epidemic is not the first time healthcare systems tried telehealth options to control the outbreak, and will not be the last one. Advantages of a successful telehealth service execution are a rapid distribution of providers, easier triage of patients, helping overloaded medical centers and staff, and reducing the risk of communicable diseases like COVID-19, contracted by close and person-to-person contacts. However, there are various obstacles in its way.^3^ Low willingness and acceptance of healthcare workers toward telehealth, lack of funding in this area, and lack of necessary organized networks and foundations are the main barriers.^34-36^ Internet-based surveillance systems are modern methods of rising public health outbreaks like COVID-19.^1^ The present study shows the installation of such a system can relieve public health concern and alongside diagnosing suspected and high-risk groups of the population, helps to lower panicked healthy people visiting medical centers and emergencies. Right now, other research teams all around the world are working on developing practical platforms offering self-screening and education options, like the framework for identifying regional outbreak and spread of COVID-19 form online population-wide surveys introduced by Rossman and colleagues,^14^ the COVID Symptom Study ongoing in the United Kingdom,^37^ and COVID-19 screening tool powered by Apple and offer services in the United States of America,^38^ and all trying to control the current terrible pandemic. One international consortium for tracking coronavirus health status is also developing and connecting all these facilities globally to help defeat the brutal coronavirus sooner and safer.^39^

 To the best of our knowledge, this is the first mass population-wide screening survey publishing from Iran. The results of this study can be used to study the features of the COVID-19 epidemic in Iran. A combination of self-screening, education and registry system programmed on this platform makes it a unique and valuable means of controlling the outrageous condition of the COVID-19 epidemic in this country. We intended to implement simple statistical analyses and methods on the collected data of participants to investigate and transfer the messages of the survey uncomplicatedly. The major limitations of the present study are patients’ privacy and data security issues. We guarantee that only the research team has access to survey data, although participants submitted no identification information on the platform. Another limitation of the platform was operating only in the Persian language and some people may have difficulty answering questions because of having another language as a mother tongue. Older age groups may have lower utilization of and trust in internet-based healthcare services. Therefore, we suggest more educational and cultural changes to support all people to use such beneficial tools during health emergencies like pandemics and also other times of non-crisis.

## Conclusion

 This study proved that implementing a proper online self-screening tool could mitigate population panic during wide-spread epidemics like COVID-19 and relieve massive influx to medical centers. Also, an evidence-based education platform can help healthcare authorities to fight more effectively against intimidating infodemic happening alongside epidemic and pandemic. Remarkable penetration and utilization of such a platform in the general population and its verified effectiveness once more validate the potency of crowdsourcing data in controlling public health catastrophes.

## Acknowledgment

 This work was funded by the National Institute of Medical Research Development (NIMAD), Tehran, Iran by grant (No. 991751).

## Ethical issues

 This study was approved in the ethics committee of National Institute for Medical Research Development (NIMAD) of Iran by the approval ID: IR.NIMAD.REC.1399.001.

## Competing interests

 Authors declare that they have no competing interests.

## Authors’ contributions

 FF designed the study. SA, EM, and KJ did the literature search. FF, SSM, EG, and MA processed the data and did the statistical analysis. NF, HZ, AM, BD, and PF developed and implemented the self-screening algorithm and website. AN and HE prepared the COVID-19 registry data from MoHME. FF, SA, SSM, and NR interpreted the results and prepared the first manuscript. All of the authors commented on the primary manuscript. FF, SA, SSM, NR, HJ, MMG, SN, BL, and RM revised the final manuscript. FF was the principal investigator of the study and superintended all phases of the survey.
